# Inequalities and Inclusion in Exercise Referral Schemes: A Mixed-Method Multi-Scheme Analysis

**DOI:** 10.3390/ijerph18063033

**Published:** 2021-03-16

**Authors:** Emily J. Oliver, Caroline Dodd-Reynolds, Adetayo Kasim, Dimitrios Vallis

**Affiliations:** 1 Department of Sport and Exercise Sciences, Durham University, Durham DH1 3HN, UK; 2 Durham Research Methods Centre, Department of Sport and Exercise Sciences, Durham University, Durham DH1 3LE, UK; caroline.dodd-reynolds@durham.ac.uk; 3 Durham Research Methods Centre, Department of Anthropology, Durham University, Durham DH1 3LE, UK; a.s.kasim@durham.ac.uk; 4 Durham Research Methods Centre, Durham University, Durham DH1 3LE, UK; dimitris.vallis@durham.ac.uk

**Keywords:** physical activity, exclusion, health, community-based, prescription

## Abstract

Physical activity prescription, commonly through exercise referral schemes, is an established disease prevention and management pathway. There is considerable heterogeneity in terms of uptake, adherence, and outcomes, but because within-scheme analyses dominate previous research, there is limited contextual understanding of this variance. Both the impact of schemes on health inequalities and best practices for inclusion of at-risk groups are unclear. To address this, we modelled secondary data from the multi-scheme National Referral Database, comprising 23,782 individuals across 14 referral schemes, using a multilevel Bayesian inference approach. Scheme-level local demographics identified over-sampling in uptake; on the basis of uptake and completion data, more inclusive schemes (*n* = 4) were identified. Scheme coordinators were interviewed, and data were analyzed using a grounded theory approach. Inequalities presented in a nuanced way. Schemes showed promise for engaging populations at greater risk of poor health (e.g., those from more deprived areas or of an ethnic minority background). However, the completion odds were lower for those with a range of complex circumstances (e.g., a mental health-related referral). We identified creative best practices for widening access (e.g., partnership building), maintaining engagement (e.g., workforce diversity), and tailoring support, but recommend changes to wider operational contexts to ensure such approaches are viable.

## 1. Introduction

### 1.1. The Positioning of Physical Activity Prescription with Respect to Inequalities

Physical inactivity is a global public health priority [1]. Targeting increasing activity levels of the most inactive is vital; the modelling of data from over 100 countries has confirmed the association between activity inequalities and health outcomes, and, importantly, demonstrated that activity inequality-centric interventions may have up to four-times more potency for reducing disease prevalence than population-wide (universal) approaches [2]. Despite this, there is limited exploration of common approaches to physical activity promotion (policies or services) through an inequality lens.

Physical activity prescription, most commonly through exercise referral, is an established model of disease prevention and management. Referral-based schemes were first established in the 1990s, and operate internationally. Typically, individuals are referred by a healthcare practitioner (e.g., from primary care) to supervised exercise provision and behavior change support, delivered by trained practitioners based in community assets (e.g., leisure centers). In contrast to more universal approaches (e.g., physical activity guidelines and healthy lifestyle campaigns), exercise referral schemes typically align with proportionate universalism [3]. That is, while increasingly accessible to all (e.g., through self-referral), they strategically target those with either poorer health or who are at risk of poorer health (e.g., pre-diabetes, fall prevention). It is therefore expected that they would be of greater benefit to those with greater health needs.

However, one of the challenges of both universally applied and targeted interventions is that those with more assets or resources within that population are better placed to take advantage of it. For example, it is reasonable to anticipate that those with greater literacy, confidence, social support, and funds to travel to and from facilities may be more likely to engage in, adhere to, and benefit from such interventions. If this is the case, then inadvertent intervention-generated inequalities can emerge [4]. Presently, it is unclear if this is the case for exercise referral, an issue recognized in national policy [5]. Named evidence gaps include comparing the effectiveness of schemes for different groups and understanding factors that encourage or inhibit participation and completion by underrepresented groups, specified as including people from black and minority ethnic groups, people with disability, and those from lower socioeconomic groups. 

### 1.2. Inequalities in Uptake, Adherence, and Outcomes 

Data exploring patterns of uptake, adherence, and outcomes of physical activity referral schemes are predominantly descriptive and focused on single schemes, though some of these are substantial [6]. Across these studies, there are some consistent predictors of outcomes. For example, increased uptake is associated with being female or older [7,8,9,10], and adherence with being male or older [6,7], or on a physical rather than a mental health referral pathway [11]. Limited work has suggested that there may be differences in uptake and adherence based on the health condition and/or disability status [9,12,13], with higher rates for musculoskeletal and cardiovascular than other forms of referrals. There is some evidence that those with higher initial levels of physical activity are more likely to uptake and adhere to schemes [13,14]. In terms of outcomes, there is some evidence that men and older adults benefit more [15]. 

There is a substantial body of predominantly qualitative work from the patient or participant perspective, highlighting the range of psychosocial factors that can explain these observed inequalities. For example, one review (of 33 studies) highlighted that social support from providers, other attendees, and family, as well as the variety and personalized nature of sessions, supported adherence [16]. Conversely, inconvenient session timings or location, cost, dislike of gym-based delivery environments, and a lack of confidence undermine adherence. In addition, a more recent review (of 24 studies) summarized a range of motivational and cognitive predictors of adherence, including intrinsic motivation, psychological need satisfaction (cf. self-determination theory [17], self-efficacy, and lower expectations for change [18].

Findings related to the influence of socioeconomic status or deprivation are more equivocal. For example, increasing deprivation was associated with greater adherence in one large (*n* > 2000) scheme [8], whereas a study of over 300 General Practice surgeries found no relationship between deprivation status and uptake or adherence [19]. General Practices within areas of deprivation were more likely to refer patients to exercise referral schemes than practices in more advantaged areas, suggesting that schemes could therefore reduce health inequalities by facilitating access to care [19]. In contrast, recent substantial work explored rates of referral and uptake in a national scheme over a nine-year period (2008–2017) [12]. Retrospective data linkage demonstrated that those in the most deprived groupings had lower uptake, with a decrease over time in referrals, and uptake rates among the most deprived relative to those in the least deprived group. Proxies for deprivation (e.g., lacking own transport) have also been previously linked to reduced uptake and adherence. 

In summary, work focused on predictors of the use of and benefits from exercise referral schemes presents a mixed picture for impacting health inequalities. While some disease groups and older adults appear to be engaged and retained by a range of schemes, those with markers of disadvantage, such as mental health conditions or lower initial physical activity levels, face challenges to participation. Importantly, an understanding of how scheme practices might impact inclusion or exclusion is limited by the dominance of within-scheme studies and minimal mixed methods work, drawing together quantitative and qualitative data to understand patterns in participation.

### 1.3. The Case for Wider Contextualised Analysis across and between Schemes

There have been calls to better understand how to support adherence to inform guidance and tailor schemes to meet participant needs [11]. While some researchers have argued that adherence to schemes should be promoted by focusing on participants’ expectations and beliefs when entering the scheme [18], we argue here that this is likely to be insufficient and unrealistic, for two key reasons.

First, many of the psychological variables important for adherence (e.g., agency and autonomy) have environmental and structural determinants. While practitioners can impact efficacy in context, qualitative work has shown that practitioners may not be adequately resourced (time or expertise) to support participants dealing with wider and more complex circumstances and needs [20]. Organizational, scheme-level, or system adaptations may be required. At the scheme level, influencing factors for uptake and adherence have been mapped onto the socio-ecological model, reinforcing the importance of wider organizational and system determinants [21]. Compared to individual-level factors, to date, these are underexplored, resulting in a limited contextual understanding of observed inequalities (i.e., what works for whom and under what circumstances [22]).

Second, as there is considerable heterogeneity between schemes in terms of both delivery (e.g., length, delivery venues, staff contact time, content) and monitoring (e.g., variables measured, measurement tools, timing of data capture and follow-up) [10,14], it is challenging to make best practice recommendations for practitioners and scheme coordinators [23]. Emerging scheme comparisons do show evidence of differences in scheme outcomes, and that longer schemes are more effective [14]. This is only useful, however, if we can support participants to adhere. To date, detailed between-scheme comparisons to identify and critique best practices for supporting adherence are lacking. In particular, it is unclear how physical activity prescription schemes can best engage and retain participants facing barriers to inclusion and health. 

### 1.4. Research Aims 

To address these gaps in understanding, we used data from the National Referral Database [24] to analyze and contextualize inequalities across, and, for the first time, between exercise referral schemes. Formed in response to the National Institute for Health and Care Excellence’s (2014) call for a national system collating local data to inform policy, commissioning, and practice [5], the National Referral Database is the largest multi-scheme exercise referral dataset currently available, comprising individual and scheme-level data from 2011 onwards [24]. This research integrated quantitative and qualitative data to understand both who exercise referral schemes do and do not work for and why. Phase 1 modelled national referral data to examine predictors of uptake (i.e., starting), adherence (i.e., completing), and outcomes (i.e., benefitting). Contextual understanding was generated by using local population demographics to identify over- and under-sampling by schemes, with high-performing schemes in terms of their engagement of and/or benefits for population subgroups at risk of poorer health identified for in-depth analysis. Phase 2 comprised qualitative interviews with coordinators of identified schemes, to facilitate deeper exploration of the components and approaches of these more inclusive schemes. The resultant grounded theory generates insight into both inclusive practices and the wider contextual influences impacting scheme delivery.

## 2. Materials and Methods—Phase 1

### 2.1. Data and Sampling 

The National Referral Database is an observational, longitudinal dataset curated by ukactive (UK-wide professional member organization), Refer-All (a company providing software solutions for exercise referral), and the National Centre for Sport and Exercise Medicine. The sampling and data collection procedure is documented fully elsewhere [24]; briefly, it comprises submissions of individual-level referee data clustered by scheme. Participant information was de-identified and uploaded into the national database by scheme coordinators or practitioners. Of note, the database contains a range of scheme types, including those commissioned and funded by local authorities and private providers. 

The data slice used for the present study originally consisted of data from all participants at January 2019, with a total of 24,086 individuals across 19 exercise referral schemes. Data cleaning, including removal of schemes with a very low number of observations, is detailed elsewhere [24]. The resultant data set for analysis comprised 14 schemes covering 23,782 participants. Though the database provides limited information on scheme characteristics, available data indicated that schemes ranged from six weeks to three months in length, and in size from 1264 to 4574 participants. The majority were based in South East England (*n* = 9), with three in North West England, one in South England, and one in South West England.

All participants were included for baseline analyses; sample sizes varied for outcome variables, as these differed by scheme. Differential non-completion was one of the data quality aspects analyzed. The protocol for this research was registered on the Open Science Framework [25]; any subsequent deviations are reported below, and pertain to insufficient data quality for intended analyses (e.g., to test our hypotheses regarding disability and to use informative priors). 

### 2.2. Analyses and Data Processing

Participant postcodes were matched to a publicly available index of multiple deprivation (IMD) data [26]. This creates an area-based index of deprivation using employment, education, and economic deprivation data. We also used the accompanying IMD decile score that divides areas into deciles nationally for deprivation rating (1 = most deprived, 10 = least deprived).

Descriptive data at the whole sample and scheme levels for (i) engagers (those who started the scheme) and (ii) completers (those with pre- and post-data) were examined for theorized determinants: age, gender, ethnicity, disability status, deprivation index, presystolic blood pressure, employment status, caring responsibilities, baseline mental well-being, body mass index (BMI), and baseline physical activity. Categorical variable constraints were pre-determined by database construction. As participants were nested within schemes, a multilevel (hierarchical) model with random intercepts was used to account for group (scheme) effects. 

To examine scheme uptake (i.e., starting), a series of logistic models within a Bayesian framework were implemented for the following binary dependent variables: Female/Male; (Age ≥ 35)/(Age < 35); Eth.Minority/White; (IMD ≥ 6)/(IMD < 6), in each case using dummy variables for each of the referral schemes as covariates in order to indicate over/under-representation within schemes compared to local demographics. The models were implemented within a Bayesian framework with vague priors; we opted for vague over informed priors to allow the data to guide the estimates. 

To examine adherence (i.e., completion), a logistic model was used with a dependent variable of Completer/Non-Completer and all relevant covariates (see Table 1) in order to measure the odds contribution of each characteristic in the determination of completion, using estimated posterior probabilities. In addition, a hierarchical model was used to define the extent to which baseline socio-demographic characteristics predicted changes in levels of physical activity of completers. Significant individual predictors were identified using posterior probabilities of the beta coefficients at the 95% high-density interval. The model was implemented within a Bayesian framework with vague priors; we opted for vague over informed priors to allow the data to guide the estimates. 

To examine outcomes (i.e., effectiveness), the primary outcome variable was the change in METmins data (MET = metabolic equivalent of task, a unit of energy expenditure; whereby one METmin represents 1kcal/kg/hour; METmins = amount of energy expended during a minute) pre- to post-participation, recorded at scheme attendance. The percentage change was rejected given the number of zero values at baseline. METmins are derived from the International Physical Activity Questionnaire (IPAQ) short form [27], which is a self-reported physical activity recall over the previous seven days. Seven questions are asked regarding activity and sitting time (frequency and duration over seven days engaged in walking, moderate and vigorous activity, and sitting). An overall METmin score is obtained by multiplying weekly minutes spent in each activity category by 3.3 (walking), 4.0 (moderate), and 8.0 (vigorous) METs. Multilevel linear regression was used to predict the change in physical activity pre- to post-participation based on a Bayesian framework. Predictors were age, markers of health risk (e.g., body mass index (BMI) category [28], IMD), and baseline physical activity level. Credible intervals were used to interpret significance.

The database includes referring professional but, importantly, not the reason for referral. As such, we constructed dummy proxies as covariates for some hypothesized determinants. Specifically, BMI was used to construct obesity classification (i.e., obesity classes I, II, III), and a mental health-based referral was composed using the referrer type as the proxy and using the indicators: “Clinical Psychologist”, “Community Mental Health Worker”, “Community Psychiatric Nurse”, “Consultant Psychiatrist”, “Counsellor”, “Mental Health Nurse”, “Mental Health Practitioner”, “Mental Health Worker”, “Mental Health Support Worker”, “Occupational Therapist”, “Psychiatrist”, “Psychologist”, and “Psychotherapist”. We also constructed a proxy covariate for leisure time using employment status markers of retired, student, and looking after home/family. 

The advantages of the methods outlined above over traditional frequentist approaches are that they allow for a more intuitive interpretation of estimates, since a probability distribution of values is provided instead of a confidence interval range. We provided credible intervals of the estimate at a 95% probability (high-density interval).

## 3. Results—Phase 1

### 3.1. Inclusion-Related Descriptives 

Whole sample baseline descriptives are published elsewhere [14]. Here, we focus on demographics of particular relevance to health inequalities and inclusion. Our initial analysis identified problematic data quality for several variables of interest; this is unsurprising, as the comprehensiveness of the database entries is shaped by local practices, yet re-affirms the challenges of collecting comprehensive evaluation data in this setting. For example, only 17.5% of participants (*n* = 4171) had an entry for disability status. Of those that did respond, 947 (22.16%) declared a disability, compared to a UK population average of 18%. Given the nature of exercise referral schemes (i.e., most will enter the scheme via a health-related referral), we might expect this to be higher. Responses were predominantly comprised of those responding mental health condition (*n* = 212), long-term health condition (*n* = 266), or other (342). 

The variable ethnicity included the response category unknown, which we converted to a missing value; 13% of participants had missing data for this variable. Across the sample, participants identified as: White (30.2%; UK population = 85%), Black (23.6%; UK population = 3.3%), Asian (15.4%; UK population = 7.5%), Mixed (2.4%; UK population = 2.2%), and Other (2.4%; UK population = 1.0%). Given that Black and Asian participants are typically underrepresented in published evaluations of exercise referral schemes (and non-targeted health interventions generally), the present sample represents a useful opportunity to explore how schemes work for these groups.

In terms of wider social circumstances, only seven participants were recorded as being carers (despite representing approximately 10% of the UK population). Over 70% of participants were from the 50% most deprived regions of the UK; 32% of participants in the sample were from the 20% most deprived of areas in the UK. Specifically, across schemes, recruitment of participants by index of deprivation decile was as follows: 1 = 2491 (10.5%); 2 = 5237 (22.1%); 3 = 3977 (16.8%); 4 = 3003 (12.7%); 5 = 2778 (11.7%); 6 = 2350 (10%); 7 = 1384 (5.8%); 8 = 1199 (5.1%); 9 = 770 (3.3%); and 10 = 496 (2.1%) (note, a higher deprivation decile represents less deprivation). At this stage, it is important to note that the data do not indicate whether schemes are recruiting participants from minority ethnic backgrounds or deprived areas particularly well, or whether they are simply representative of their local demographics.

Lastly, indicators of entry-level health, systolic blood pressure, and well-being also showed high rates of missing data, most likely due to those variables not being part of the specific scheme’s evaluation outcomes, or captured using different forms of assessment. The poor quality of well-being, quality of life, and mental health-related data led us to use a proxy variable for mental health referrals based on referrer type, as outlined above. Due to the database type, it is unknown whether the data is missing non-randomly, an issue we return to in our discussion.

### 3.2. Model 1: Scheme Uptake

Over- and under-representation in uptake was identified by comparing scheme-level data to local demographics drawn from adult census data (see Supplementary Tables S1–S5). Results (reported hereafter, and in Table 1, as odds ratios with 95% credible intervals) demonstrated a consistent overrepresentation of women (DV = female, lowest probability of equal representation = 0.51 [0.38–0.73]) and older adults (DV = above 35; lowest probability = 0.81 [0.76–0.85]). Those from areas of deprivation (DV = high deprivation = 0.93 [0.93–0.94]–0.12 [0.04–0.22]) or minority ethnic backgrounds (DV = Eth.Minority = 0.76 [0.72–079)–0.01 [0–0.03]) were also overrepresented, though with more variation in the degree of overrepresentation between schemes. 

### 3.3. Model 2: Scheme Completion

Logistic regression was used to examine the probability of scheme completion, initially without interaction terms. Two schemes (5115, 5026) were omitted due to lack of observations, and a further three (5002, 5131, 5119) due to collinearity. The model converged after 10,000 iterations. The results (see Table 1) indicate that higher age (0.02, 95% credible interval = [0.02, 0.03]), being female (0.08 [−0.01, 0.016]), having more leisure time (0.15 [0.02, 0.27]), and belonging to a higher IMD decile (lower deprivation; 0.02 [0.00, 0.04]) all increased the odds of completion. Conversely, a mental health referral (−0.71 [−1.07, −0.36]), increasing obesity (CL1: −0.04 [−0.13, 0.06], CL2: −0.08 [−0.19, 0.03], CL3: −0.25 [−0.37, −0.13]), and belonging to an ethnic minority group (−0.03 [−0.10, 0.05]) lowered the odds of scheme completion. A further model with interaction terms added to control for individual group effects for gender, ethnic minority status, and age (≥35 years) converged after 100,000 iterations, but identified few additional significant effects (see Supplementary Data).

### 3.4. Model 3: Scheme Outcomes

A baseline model identified that, across schemes, the average change in METmins was 1371 with a 95% credible interval of [1307.3–1429.1]; this indicates a 95% probability that the average change in METmins across schemes is different from zero. A multilevel model using vague priors converged after 100,000 iterations (see Table 2). The results indicated that higher age (−5.17 [−8.71–1.79]) was associated with increased odds of a positive change in physical activity levels. We noted a highly significant coefficient for age. Pre (i.e., baseline) systolic blood pressure was also significant and positive, that is, higher baseline values will lead to a positive change in METmins. 

## 4. Materials and Methods—Phase 2

### 4.1. Sampling and Recruitment 

Ethical approval was obtained from the Department of Sport and Exercise Sciences, Durham University. Schemes were purposively selected on the basis of Phase 1 data and on the basis of their relevant insight to the topic under study, a process typical in grounded theory [29]. Specifically, the best performing schemes in terms of engaging and/or outcomes for underrepresented groups were identified from the data, with the rationale discussed by the research team; researchers were blind to the scheme identity during selection. Scheme codes were submitted to the database owners, who contacted relevant scheme coordinators with an invitation to participate. Four of the five scheme coordinators approached agreed to participate, received an information sheet, and provided informed consent; an overview of participating scheme details is shown in Table 3, in a manner that maintains scheme anonymity.

### 4.2. Data Collection and Analysis 

Participants engaged in a remote (virtual) one-on-one interview with the first author that followed a semi-structured interview guide developed in line with recommendations [30]. The interview focused on three key areas. First, the delivery context was explored to allow participants to describe their roles and schemes, as well as enabling us to develop an understanding of how organizational, structural, and environmental (e.g., local area) characteristics may impact inclusion-related challenges and practices. Second, data from Phase I relevant to the scheme was discussed with the coordinators, focusing on their understanding of how inclusion might present in their scheme alongside proactive or responsive practices. Third, the interview focused on future plans for inclusive practice within the scheme. All interviews were recorded; written summaries were sent to participants after their interview to identify any areas for further discussion, and one participant sent through documents to add contextual understanding. 

**Figure 1:**
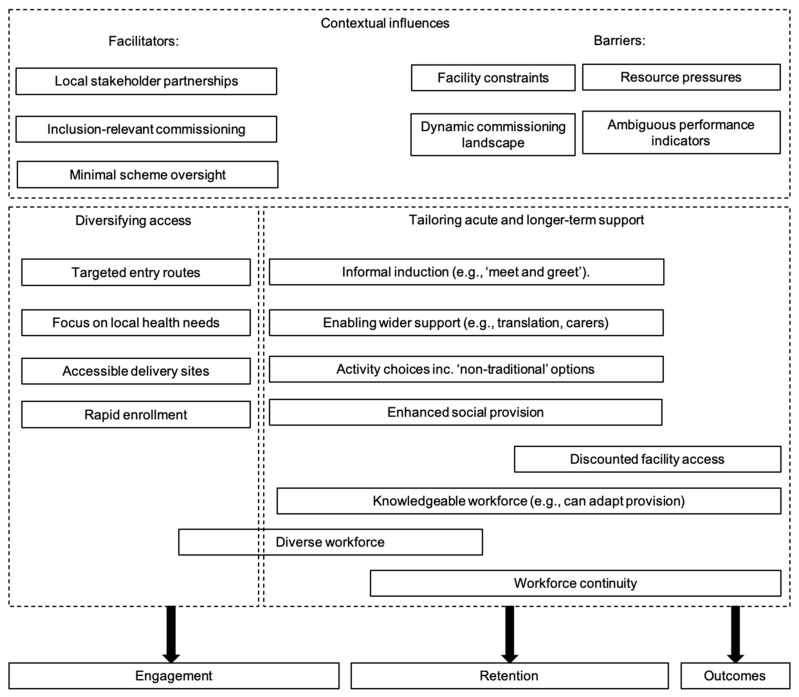
Conceptual schema of inclusive practices in exercise referral schemes.

The qualitative data were analyzed using a grounded theory-based approach common to health services research [31]. Data were analyzed through constant comparison between and across interviews, coding data for both concepts (e.g., practices for inclusion) and relationships between them (e.g., how inclusive practice is supported) [29,32]. During the data collection process, quotes relevant to the research question were highlighted and clustered into four parent codes. These initial organizational codes were labelled as diversifying access, tailoring support, supporting long-term engagement, and contextual influences, guiding the probing for detail in these areas in subsequent interviews. Data relating to context informed latter-stage coding focused on developing theory about contextual constraints on and contextual facilitators of inclusive practices. We found it helpful to organize our codes into a conceptual schema to summarize our findings (see Figure 1). The components of the framework are presented in narrative form with integrated interview data. 

## 5. Results—Phase 2

### 5.1. Diverisfying Access 

Participants described a range of ways in which their schemes encouraged and enabled a wider range of individuals to access their services. Predominantly, these were via the diversifying of traditional entry pathways (e.g., via General Practice (GPs) or clinical settings) or delivery sites (e.g., leisure). In terms of entry pathways, for example, one participant noted that “We’re seeing a lot more charity-based [referrals] and also interestingly we’ve seen an increase in private physios referring to us” (S1). Schemes reported partnerships with social prescribers, local charities, and stakeholder groups (e.g., “Our local mental health group”; “We have regular think-tanks with healthy community managers”), which were facilitated in some cases by co-location (e.g., “We’ve got a physio[therapy] centre in one of our sites”). Schemes reported the importance of dedicated staffing for building and maintaining these relationships:

*“My role is to go out and look at different ways to bring referrals in. So I spend a lot of time going off to forums, umm, health task and finishing groups and CCG meetings to see kind of what’s going on out there and try to bring stuff back into the centres.”*

Multiple schemes noted the importance of “rapid contact” post-referral, with one making “a conscious effort to get them [referees] booked in earlier ... we noticed an increase in first assessment [attendance]” (S4). One scheme operated through a centralized referral hub that would follow up with telephone referrals that had not made contact with schemes (S3). This was perceived to be particularly important for participants who might be hesitant or more reluctant to engage in these settings. 

Using a range of delivery venues was also seen as a way of supporting inclusion: “If you look at leisure centres programs, it’s not inclusive for everyone” (S4). Noting an issue with an older site (“We’re not on a bus route ... our number one issue is transport” (S3)), one scheme had shifted delivery of some services to community venues, including “a local hall right in the middle of the centre of town.” A different scheme had piloted delivery in an assisted living facility in partnership with a housing association. 

Importantly, widening access was informed by local health needs and priorities, rather than just seeking general expansion (e.g., “Looking at the local demand ... there’s a lot of focus on strength and balance of over 80-year-olds, so we’ll try and develop this area”; “We’re trying to generate a junior mental health referral pathway ... we’ve had issues with young peoples’ mental health in this area”). One scheme has explicit performance targets for recruitment of priority groups: “We are targeted with getting at least 70% of people in that are from deprived wards within that area” (S2). Schemes reported targeted funding to support engagement of particular groups (e.g., “We’ve done a pilot scheme with [redacted] council funding cancer patients to attend the program for free”).

### 5.2. Tailoring Support 

A number of good practices were identified in terms of tailoring support to help those who may face additional barriers to engage and adhere. These occurred throughout the referral pathway, including during enrolment/arrival, through the provision of particular activities or pathways, and for dropout prevention. A key mechanism for identifying the need for and delivering this support was the workforce team. 

With respect to strengthening initial support, on arrival, one scheme had developed a “meet and greet” process “if someone needs a little more hand-holding”: “If someone is nervous about coming to the gym on their own, we meet them by the front door, help them check in, walk them up to the gym.” One scheme used more informal “café spaces” for first assessments (S2), and another kept the initial consultation exercise-free (S1). Multiple schemes mentioned provision for supporting the engagement of clients with wider needs or barriers: “If someone has dementia and they come to the centre, we allow a carer to go in for free with them as their support” (S1); “We have had a couple of cases where we’ve had to provide a sign language person [interpreter]” S2; for participants who do not speak English, “They’re asked to bring an interpreter with them or ... a friend who can translate for them” (S2). Interestingly, one scheme had responded creatively to capacity pressures by doing joint inductions, with the unintended benefit effect of strengthening social support: “People that join together tend to become quite close ... you can see the friendships blossom, which is quite nice to watch” (S2).

There was clear awareness of the importance of social facilitators for retention: “There’s a real community feel when you come inside ... everyone is friendly, they integrate, people are open with each other. They’re enjoying each other’s company” (S2). To reinforce this, multiple schemes provided opportunities for social interaction around and outside of formal sessions. One scheme had participant-led weekly coffee mornings and a social meeting “once a month down the local garden center” that the staff attended (S4). The coordinator noted that this had seemed particularly useful “because I do get a lot referred for bereavement of isolation ... it’s quite hard to come into a group of people and say hi, do you want to get a coffee, so that way they become friends, they start talking, people hit it off and, you know, my work is done and they go off and do their own thing.” All schemes featured specific actions if a participant missed sessions, for example: “If someone hasn’t been attending and it isn’t like them ... It’s quite heavily tracked if people are not attending.”

Across schemes, coordinators recognized the importance of the staff team for supporting a range of participants to engage: “I think it’s because, as a rule, we keep the same staff ... people get to know people ... our staff know people by names ... rather than a large leisure centre, where you lose that personal touch” (S3); “We’ve had members of staff that started at the beginning of the program who are still working on the program” (S1). The benefits of continuity and accrued experience were emphasized, with staff who have “been doing it for years and years”, so that coordinators can “trust their judgement” in terms of tailoring support. Of relevance for inclusion, scheme coordinators commented on the diversity of their staffing teams: “On our staff we do have a mix. It’s absolutely crucial” (S2); “We’re quite lucky with the team that we have; it is a multi-cultural team who speak a lot of languages” (S4); “We have a mixture of staff, we do have some youngsters that are early twenties, right up to, you know, forties, fifties ... and we have quite a diversity, so we have a deaf lady at one of the sites as well, we have a mixture of male and female staff, which sometimes makes a difference if you have a male for young men, for example” (S1). Coordinators felt that this particularly contributed to the uptake and retention of ethnic minority participants: “You don’t feel like an outsider ... the worst thing is if they come in no one speaks their language”. 

Lastly, support was tailored via enabling participant choices. For example, schemes offered “interim chats ... in whatever form suits the patient” (S1) or “the option for people who are lower risk to come in at a range of times, which makes it a bit freer for them” (S2). All schemes were keen to diversify provision: “We’ve adapted it ... to try and diversify what we offered a little bit ... to get away from the old conventional exercise referral in a gym” (S2). Two schemes linked available activities to their ability to support target groups in particular. One noted that the provision of water-based pain relief was particularly popular with men: “Because we tailor what we offer, that’s possibly why we see more men” (S4). A second noted that partnering with private health suite spas had supported retention rates for participants with mental health referrals in particular, as “mentally, they can relax as well as physically” (S1). 

### 5.3. Long-Term Engagement 

Though long-term effectiveness was not a focus of this research, coordinators did comment on relevant mechanisms for supporting ongoing engagement that may benefit those facing barriers to engagement. All schemes included some form of subsidized membership or reduced facility rates post-scheme: “We have a specific membership for GPs [referrals] to go onto, which is something like £20 per month less than the standard one, of which they can stay on that up to a year” (S1); “After the final assessment, they’re offered a discount[ed] membership” (S4); “When people do complete, they have the option of subsidized membership ... and there’s a pay-as-you-go option, which is heavily subsidized, I think down to £2 [a session] ... it just makes it a bit more inclusive” (S2). Schemes also linked discounts to certain criteria (e.g., “There’s a lower price if they’re over a certain age”); one scheme noted that “If they’ve got a long-term condition ... we generally don’t fiddle with it [the discounted membership] ... some of them have been on it for fifteen years” (S1). 

### 5.4. Contexutal Challenges 

This category concerned factors impacting the ability of schemes to operate inclusively. These included facility constraints, resource pressures exacerbated by untargeted or complex referrals, and fluctuating and somewhat ambiguous commissioning landscapes.

Several schemes reported issues with finding appropriate spaces for delivery (e.g., “We are limited by our space and now losing a venue ... they are shutting for a refurb. We’re now looking for other venues, because we want to keep these classes in the community” (S3)). One scheme that used predominantly dual-use (i.e., school and leisure) venues noted this was “very tied up”. This fed into a wider capacity issue for schemes; “The main challenge is the total amount of referrals that are coming in ... it’s a very high number” (S2). While scheme coordinators wanted to be inclusive (e.g., “I don’t say no to anyone” (S1)), this untargeted approach (e.g., “We seem to take everything, I’m not sure why.”) led to compromises in delivery (e.g., “Because there is such a high volume, a lot of the time is spent on initial assessments. In their quieter sites, you can do more reviews” (S2); [on following up missed attendances] “There’s no more I can do because I’ve spent a lot of time chasing this guy and I’ve still got X amount of people to sort out” (S3)). Resource pressures were explicitly cited as the reason schemes could not provide sessions to meet an identified need (i.e., a quieter mental health hour; women-only sessions).

Schemes also faced challenges supporting referees with complex health needs. Examples were provided in terms of mental health referrals (“A nightmare ... we’ve had one lad referred to me four times over the years. I said this is the last time, I can’t keep doing this, and he looked so much better, said yes yes every time I phone, I email, I text, I call him, eventually he’ll answer we’ll book an appointment then ... cancels” (S3)) and those with more complex physical needs or conditions: “Two we weren’t able to work with, they were just too complex, we said no there is absolutely nothing we can do”; “We can’t deal with their physical needs ... sometimes their needs are quite complex and we just don’t have the facilities to manage that safely” (S1).

Though not specifically related to inclusion, scheme coordinators spoke of the challenges of operating within a dynamic commissioning landscape. One described how “as the program shrunk and the value wasn’t put on physical activity as much by the GPs, we lost staff ... Now they’re starting to see a value ... which means our program is growing again” (S1). Scheme coordinators felt that direction from commissioners was unclear: “Originally, they gave us notice that they were going to end everything. Then I think they got some kick back ... they’ve decided to continue with cardiac referral and stroke [pathways]” (S3); “All through the contracts, the council has always insisted the ERP had to be there, but didn’t really give any parameter how small, how big, etc. We just had to provide this service ...” (S1). Funding confirmations could be for short-term periods only (e.g., “a further year extension”). Collectively, these factors made long-term planning challenging.

### 5.5. Contexutal Facilitators 

Despite wider challenges, coordinators described ways of working that have facilitated inclusive practices. For example, coordinators noted that health service developments forming partnerships between GP surgeries had led to sharing of good referral practices (“Where one was doing a better job, the others have now picked up on the process” (S1)). Coordinators also reported forming informal partnerships between schemes, involving cross-referral if this would support clients with specific barriers to continue to engage (e.g., more accessible location; more affordable services). It was noted that this might be controversial from a business perspective: “I don’t know what my managers would think about that!” (S2). 

This willingness to act in the best interests of participants was apparent within schemes too; when discussing how to support participants facing financial challenges, one participant noted “If someone was struggling, I would look at an option for them ... if it’s a financial thing, we’d work out something for them.” It was apparent that some of these practices were able to occur due to the manner in which schemes tend to function relatively independently of oversight on a day-to-day basis: “Our systems don’t really allow it, but ... I don’t have much power, but I use every inch of it”. 

Lastly, with respect to commissioning, there were some helpful practices discussed relating to inclusion of key performance indicators for targeted groups (e.g., deprivation; mental health conditions), which in turn led to specialized training (“Our staff actually had mental health awareness training as well, so they knew how to deal with people better ... To try and give them a bit of knowledge about what to expect.”). 

To draw together our codes and demonstrate relationships between factors (in a manner common to grounded theory work [33]), a conceptual schema of the findings is presented in Figure 1.

## 6. Discussion

### 6.1. Overview of Findings

This research explored inequalities in uptake, adherence, and outcomes of UK-based exercise referral schemes, and developed an understanding of practices that may help or hinder inclusion. Four key over-arching findings emerged. First, inequalities demonstrated notable homogeneity across schemes. For example, over-recruitment of females and older adults relative to local demographics was pervasive. Second, observed differences in uptake and adherence in particular are likely to have nuanced impacts on health inequalities. While there is some evidence that exercise referral schemes can engage groups known to have higher risk of poorer health, completion odds are adversely impacted by complex or challenging circumstances (e.g., deprivation). Third, data quality for inclusion-related variables (e.g., disability) was notably poor; this lack of reporting may imply something important about the historical prioritizing of this data by schemes and commissioners. In addition, large unexplained variance in outcomes suggests that unobserved covariates (e.g., facility type) are also likely to be important. Fourth, where scheme differences of interest did emerge (e.g., better retention of participants from ethnic minority backgrounds), coordinators reported a range of creative and targeted methods of widening access and tailoring support. It was noted, however, that such approaches are influenced by broader commissioning contexts and service pressures. These findings are considered in greater depth below.

### 6.2. Observed Inequalities in Uptake, Completion, and Outcomes

The importance of observed patterns of uptake, completion, and outcomes across schemes is challenging to fully elucidate, but can be considered with respect to the likely health needs of demographic subgroups. We would expect and indeed target differences in uptake, given well-established inequalities in health. For example, we would anticipate that older adults, minority ethnic participants, and those from deprived areas would be more likely to have poorer health [34], and therefore more likely to be signposted into referral schemes. The observed increased odds of participation of these groups in our data suggests this is the case, that exercise referral schemes are engaging those likely to have poorer health, and thus have the potential to positively impact health inequalities. 

The observed over-recruitment of females is more challenging to interpret. Though men have an increased risk of poorer health when compared to women (e.g., lower disability-free life expectancy [35]), particularly in diseases dominating referral routes (e.g., coronary heart disease), there are some notable exceptions (e.g., dementia [36]). Women also are more likely than men to be inactive [37]. One interpretation is that those making referrals are prioritizing inactivity as an indicator of need. However, existing work [13] showing that participants in such schemes are typically moderately active would suggest that this is unlikely. Our interpretation is that these data are consistent with previous findings that fewer men are referred into schemes [7,8,9,10], which may reflect biases in referral practices, lower engagement with health services by men, and/or that men are less likely to attend when referred. Unsystematic referrals influenced by gender, age, and perceived motivation of patients have previously been demonstrated, exacerbated by referral criteria that make almost all patients eligible [38]. Our data would support previous conclusions that stricter targeting of the referral process is needed to ensure greater consistency of referral in targeted groups [38]. Here, where local partnerships had identified a need and agreed to target it, referrals from that population increased. To add clarity to interpretations of over- and under-recruitment into schemes, future work would benefit from integrating the available data on area-level disease prevalence alongside demographics, as well as data on the characteristics of non-attending referees (not present in the National Referral Database). 

Patterns of completion in the present data are perhaps more concerning with respect to health inequalities. Increasing age and being female increased the odds of completion, as in previous work [6,7], highlighting an existing concern in the literature that schemes are not well-suited to younger or male participants. In addition, a range of variables associated with poorer health decreased the completion odds (i.e., deprivation, obesity, mental health referral, and belonging to an ethnic minority group). This is consistent with previous research showing that complex or impaired social circumstances can undermine participants’ ability to adhere to schemes [21]. These observations are contextualized by the qualitative data that described the limitations of schemes to appropriately support clients in more challenging circumstances. Here, we note, as argued previously [23], that these data suggest a need for better triaging at the point of referral, and that resources must be redirected so that patients with complex needs receive alternative and more intensive support. Otherwise, failure experiences for patients and schemes will persist at a time when engaging at-risk groups is more critical than ever, particularly when emerging post-pandemic data identify lower physical activity levels for men, those with complex disabilities or long-term health conditions, and those from Asian (excluding Chinese) and Black backgrounds [39]. 

### 6.3. A Recurrent Issue—Data Quality and Exercise Referral Evaluation

Despite useful findings, we were unable to conduct all of our planned analyses due to insufficient data concerning some variables of interest. While the National Referral Database offers a lot to researchers in terms of between-scheme comparisons and modelling outcomes at scale, it suffers from some of the same challenges identified in scheme-level evaluations [23]. That is, the consistency and comprehensiveness of its source data are problematic. This is an understandable outcome of processes that rely on data collection by practitioners in resource-pressured contexts; ongoing work to develop the database or to provide feasible standardized reporting tools and taxonomies [40] is helpful in this regard. Given the focus of the present study, we raise concerns about the poor data quality concerning inclusion-related variables in particular (e.g., disability, ethnicity), which makes identifying and evidencing inequalities challenging. Data sets that have sufficient power to explore changes in inclusion over time are emerging [12], but these have yet to be used to compare scheme practices in depth, a clear area for future development. 

To some extent, scheme practices, including reporting, must be driven by wider policy or structural requirements. In our interviews, only one scheme coordinator discussed inclusion or health inequality-focused performance indicators as part of their commissioning contract. Though the use of performance indictors requires caution to avoid unintended negative consequences [41], we suggest that greater contextualized monitoring and reporting of performance relative to specific targeted groups would be beneficial for driving more focus and resources within schemes toward these groups. Schemes that are incentivized on the basis of percentage completions or overall numbers engaged are unlikely to prioritize harder-to-reach clients or those with more complex needs. 

### 6.4. Learning from Inclusive Practices in Schemes

Our interview data highlighted a number of mechanisms for engaging and supporting clients with diverse needs. Promisingly, practices attempted to change or compensate for barriers at multiple levels (i.e., individual, social, environmental), demonstrating some compatibility with social and ecological models of inclusion most commonly explored in disability or educational contexts [42]. Work diversifying and strengthening entry routes for those with health needs (e.g., through working with local General Practices) or providing financial subsidies or support (e.g., discounted memberships) aligns with the neoliberal ideas of inclusion focused principally on access and economics [43]. Alongside this, work focusing on community building and empowerment of staff and participants may reflect aspirations for broader social justice-based outcomes. While, in the present study, our participants did not relate their practices to particular strategic aims or ideals, it is encouraging that the described practices are likely to target a range of known determinants of engagement.

Here, our conceptual map is intended to organize our data in a way that helps explain how practices might interrelate to both context and outcomes [33]. That, and how, the commissioning environment may constrain or support a scheme’s approaches to inclusivity is clear. While we did not observe clearly conflicting strategic and delivery-related goals, as in previous detailed within-scheme work [44], there was unhelpful ambiguity that can lead to confusion in terms of who schemes should be targeting. 

Of note, in line with established approaches to grounded theory-based work [33], it was not our intention to create, nor do we see these ideas, as fully-fledged theory. Instead, we encourage future work to critique, expand, and develop this initial understanding. While a strength of grounded theory is to reveal high-level concepts and theories not specific to specific participants or settings [45], it can lack the precision needed to fully understand links between contexts, mechanisms, and outcomes. We expect future critical realist-based work to be particularly helpful in this regard. 

### 6.5. Limitations

While we have raised a number of critical comments throughout this paper, here, we summarize the key limitations of the work. First, secondary data analysis, especially of that collected in naturalistic settings, has inherent challenges in terms of quality control and data insight. The National Referral Database was problematic in three ways in particular: the variability of submitted data between schemes, the amount of missing data at baseline and our inability to explore why, and finally the absence of variables of theoretical interest from its standardized response categories. Of note, database stakeholders have been receptive to ongoing conversations concerning additional variables of interest (e.g., the mode of delivery and referral reason), and are keen to encourage more consistent use of the database; work in these regards is ongoing. Given that the qualitative data highlighted the perceived importance of scheme-level characteristics (e.g., location, facility type, workforce), future research could usefully explore the role of these and whether they explain differences at the scheme level. It is important to note that the proportion of schemes within the database relative to those in existence is unclear [24] due to poor national data on scheme availability. The majority of included schemes are based in South East England and in urban locations; though many of these were in areas ranked highly in terms of deprivation, this bias may have implications for understanding how schemes operate in different operational and cultural contexts. Lastly, data on outcomes should be interpreted with caution, given that the completion odds were related to observed characteristics. That is, attrition biases may mean that there are systematic differences between those who complete and those who engaged initially that could affect the estimated effects on METmins change. 

A second limitation of the work is that the quantitative analytical approach treats relevant variables independently. Conceptually, this is at odds with increasing awareness of and the need to explore experiences at the intersections of identity (e.g., gender, ethnicity, and age). While the challenges of analyzing intersectionality are widely discussed [46], the constraints of the current data set prevented the application of more advanced methods of analysis to explore intersectional inequalities. Though here we were able to provide provisional data concerning demographic markers of the risk of non-engagement or non-completion, we recommend future work to explore whether there are stronger or unique effects experienced by more specific subgroups of the population. Some previous work in exercise referral suggests that risk factors may cluster, for example, that mental health referrals were more likely to be younger and unemployed [11]; future analyses may be usefully informed by focusing on groups shown to experience poorer outcomes or discrimination within wider health services. 

Lastly, we note that grounded theorists have cautioned against a reliance on single data sources (e.g., interviews), and recommend integrating multiple methods of data collection, including observations [47]. Without this, it is possible that researchers may prioritize experiences of included participants as opposed to the underlying social processes [48]. We have organized our data in a manner to emphasize the relationships between concepts, and explicitly invite future researchers to expand and develop our model. It is possible, however, that elements of our model are unrepresentative or that key limiting contextual factors are missed, given that they emerged from the experiences of scheme coordinators, whose schemes were atypical in terms of performance. 

## 7. Conclusions

We applied an inequalities-focused lens to analyze the performance and practices of UK-based exercise referral schemes, using a multi-scheme dataset collated in naturalistic settings. In contrast to previous interpretations of schemes, our data demonstrate the *potential* for exercise referral to positively impact health inequalities, in terms of schemes’ ability to engage those with poorer health or living in areas of deprivation. Local partnerships working to identify and promote access for targeted groups can strengthen this approach. Conversely, untargeted referrals pressurize scheme resources and undermine the ability of schemes to support patients with more complex needs (e.g., a mental health referral, higher obesity, living in a more deprived area) to complete, risking exacerbating inequalities. Important questions are therefore raised concerning the overall purpose of schemes and how best to target provision. 

It is clear from this multi-scheme analysis that some patients’ needs are beyond the scope of the support that may be provided by an exercise referral scheme. Some may have higher priority needs than physical activity uptake (e.g., housing, welfare, clinical); others may require more substantive support for long-term activity uptake, perhaps through priming interventions (e.g., pre-scheme telephone counselling) or personalized support similar to that provided in other behavior change areas (e.g., substance misuse). While Sport England’s (2021) strategy [49] calls for a strengthening of the connection between physical activity and health systems so that more people are referred into activity, we need to have the appropriate pathways for those with the greatest needs before we can expect positive outcomes for those groups. 

Ideally, exercise referral schemes should be targeted towards those with a clear health need, who can realistically be supported to engage if best practices are followed. Our data suggest that differences can be made at the scheme level. Identifying and sharing best practices is therefore essential to enable more participants, especially those facing challenging circumstances, to succeed. As such, we recommend both expanding data-sharing and formalizing ways of sharing best practices, or cross-pollination of ideas, across and between schemes. From a health inequality perspective, commissioners and providers need to allow for creative innovation when designing systems for physical activity uptake that triage more effectively and provide appropriate support, likely beyond traditional exercise referral schemes, for those with multiple barriers to becoming active. As a final point, we note that a physical activity prescription typifies emerging preferred models of service design and delivery that signpost individuals from primary care to community-based delivery sites and organizations. We therefore hope that lessons learnt from this examination of inequalities in exercise referral schemes inform wider approaches to public health and health service delivery.

## Figures and Tables

**Table 1:** Scheme completion (without interaction terms).

Completer (=1)	Odds Ratio	SD	2.5% (CI) *	97.5% (CI) *
Constant	−1.18	0.22	−1.62	−0.77
Female	0.08	0.04	−0.01	0.16
PreSBP	0.00	0.00	0.00	0.00
PreMetMins	0.00	0.00	0.00	0.00
Mental	−0.71	0.18	−1.07	−0.36
Leisure	0.15	0.06	0.02	0.27
Age	0.02	0.00	0.02	0.03
IMDde	0.02	0.01	0.00	0.04
Scheme 5002	−0.15	0.09	−0.34	0.03
Scheme 5144	−2.71	0.79	−4.41	−1.37
Scheme 5036	−0.40	0.08	−0.55	−0.25
Scheme 5056	−0.22	0.07	−0.36	−0.07
Scheme 5063	0.37	0.15	0.07	0.67
Scheme 5072	−0.07	0.09	−0.23	0.11
Scheme 5108	0.58	0.18	0.23	0.93
Scheme 5156	0.13	0.38	−0.62	0.88
PreHR	0.00	0.00	−0.01	0.00
EthMinor	−0.03	0.04	−0.10	0.05
PreDBP	0.00	0.00	0.00	0.00
CL1obese	−0.04	0.05	−0.13	0.06
CL2obese	−0.08	0.06	−0.19	0.03
CL3obese	−0.25	0.06	−0.37	−0.13

* Lower and upper tails of the 95% credible interval of the posterior distribution.

**Table 2:** Outcomes of scheme completion (DV = change in METmins).

ΔMETMins	Mean	SD	2.5% (CI)	97.5% (CI)
Constant	466.64	325.75	−181.06	1101.36
Female	−10.82	48.03	−105.29	82.51
Age	−5.17	1.77	−8.71	−1.79
IMDde	1.50	11.65	−21.25	24.15
PreHR	0.11	1.97	−3.71	3.98
EthMinor	50.89	45.90	−37.27	141.41
PreDBP	0.91	1.02	−1.08	2.92
PreBMI	−2.37	1.78	−5.81	1.08
PreSBP	2.61	1.36	−0.03	5.29
MentalH	−187.16	216.65	−609.99	235.07
Scheme 5144	−204.57	508.87	−1286.22	754.44
Scheme 5002	150.90	239.42	−339.79	645.42
Scheme 5036	53.35	235.43	−420.90	544.14
Scheme 5056	24.73	234.27	−441.03	515.61
Scheme 5063	−907.51	258.06	−1453.60	−409.04
Scheme 5072	261.42	236.12	−212.49	760.54
Scheme 5108	13.44	260.57	−491.35	545.85
Scheme 5156	780.09	386.59	117.82	1608.08

**Table 3:** Outcomes of scheme completion (DV = change in METmins).

ID	Location	Selection Rationale
S1	South East England; rural	Lower overrepresentation of older adults (i.e., relative to other schemes, more younger adults engaged)
S2	London Borough; urban	High completion rates for ethnic minority participants
S3	London Borough; urban	High effectiveness and completion rates across all participants (i.e., less drop-out in high-risk groups).
S4	South West England; rural	Lower overrepresentation of women (i.e., relative to other schemes, more males engaged)

## Data Availability

Access to the National Referral Database can be negotiated via contacting ukactive. Phase 2 data are not publicly available due to containing identifying data concerning the participants and schemes; de-identified transcripts can be obtained from the corresponding author upon reasonable request and with the permission of the participants.

## Supplementary Materials

The following are available online at https://www.mdpi.com/1660-4601/18/6/3033/s1, Table S1: Probability of over/under representation of females at the scheme level; Table S2: Probability of over/under representation for participants of 35 years old or above; Table S3: Probability of over/under representation of participants from an ethnic minority background at the scheme level; Table S4: Probability of over/under representation for participants with an IMD decile of 1–5 at the scheme level; Table S5: Scheme completion with interaction terms.

## References

[1] World Health Organisation (2018). Global Action Plan on Physical Activity 2018-2030: More Active People for a Healthier World.

[2] Althoff T., Sosič R., Hicks J.L., King A.C., Delp S.L., Leskovec J. (2017). Large-scale physical activity data reveal worldwide activity inequality. Nat. Cell Biol..

[3] Carey G., Crammond B., De Leeuw E. (2015). Towards health equity: A framework for the application of proportionate universalism. Int. J. Equity Health.

[4] Lorenc T., Petticrew M., Welch V., Tugwell P. (2013). What types of interventions generate inequalities? Evidence from systematic reviews. J. Epidemiol. Community Health.

[5] (2014). Public Health Guidance [PH54]: Physical Activity: Exercise Referral Schemes. https://www.nice.org.uk/guidance/ph54.

[6] Kelly M.C., Rae G.C., Walker D., Partington S., Dodd-Reynolds C.J., Caplan N. (2016). Retrospective cohort study of the South Tyneside Exercise Referral Scheme 2009–14: Predictors of dropout and barriers to adherence. J. Public Health.

[7] Pavey T.G., Taylor A.H., Fox K.R., Hillsdon M., Anokye N., Campbell J.L., Foster C., Green C., Moxham T., Mutrie N. (2011). Effect of exercise referral schemes in primary care on physical activity and improving health outcomes: Systematic review and meta-analysis. BMJ.

[8] Hanson C.L., Ellis J.G., Allin L.J., Dodd-Reynolds C.J. (2013). An evaluation of the efficacy of the exercise on referral scheme in Northumberland, UK: Association with physical activity and predictors of engagement. A naturalistic observation study. BMJ Open.

[9] Tobi P., Estacio E.V., Yu G., Renton A., Foster N. (2012). Who stays, who drops out? Biosocial predictors of longer-term adherence in participants attending an exercise referral scheme in the UK. BMC Public Health.

[10] Shore C.B., Hubbard G., Gorely T., Polson R., Hunter A., Galloway S.D. (2019). Insufficient Reporting of Factors Associated With Exercise Referral Scheme Uptake, Attendance, and Adherence: A Systematic Review of Reviews. J. Phys. Act. Health.

[11] Tobi P., Kemp P., Schmidt E. (2017). Cohort differences in exercise adherence among primary care patients referred for mental health versus physical health conditions. Prim. Health Care Res. Dev..

[12] Morgan K., Rahman M., Moore G. (2020). Patterning in Patient Referral to and Uptake of a National Exercise Referral Scheme (NERS) in Wales from 2008 to 2017: A Data Linkage Study. Int. J. Environ. Res. Public Health.

[13] Wade M., Mann S., Copeland R.J., Steele J. (2019). Effect of exercise referral schemes upon health and well-being: Initial observational insights using individual patient data meta-analysis from the National Referral Database. J. Epidemiol. Community Health.

[14] Rowley N., Steele J., Wade M., Copeland R.J., Mann S., Liguori G., Horton E., Jimenez A. (2020). Are Exercise Referral Schemes Associated With an Increase in Physical Activity? Observational Findings Using Individual Patient Data Meta-Analysis from the National Referral Database. J. Phys. Act. Health.

[15] Dodd-Reynolds C.J., Vallis D., Kasim A., Akhter N., Hanson C.L. (2020). The Northumberland Exercise Referral Scheme as a Universal Community Weight Management Programme: A Mixed Methods Exploration of Outcomes, Expectations and Experiences across a Social Gradient. Int. J. Environ. Res. Public Health.

[16] Morgan F., Battersby A., Weightman A.L., Searchfield L., Turley R., Morgan H., Jagroo J., Ellis S. (2016). Adherence to exercise referral schemes by participants—What do providers and commissioners need to know? A systematic review of barriers and facilitators. BMC Public Health.

[17] Ryan R.M., Deci E.L. (2000). Self-determination theory and the facilitation of intrinsic motivation, social development, and well-being. Am. Psychol..

[18] Eynon M., Foad J., Downey J., Bowmer Y., Mills H. (2019). Assessing the psychosocial factors associated with adherence to exercise referral schemes: A systematic review. Scand. J. Med. Sci. Sports.

[19] Sowden S.L., Breeze E., Barber J., Raine R. (2008). Do general practices provide equitable access to physical activity interventions?. Br. J. Gen. Pr..

[20] Hanson C.L., Oliver E.J., Dodd-Reynolds C.J., Allin L. (2019). How do participant experiences and characteristics influences engagement in exercise referral? A qualitative longitudinal study of a scheme in Northumberland, United Kingdom. BMJ Open.

[21] Birtwistle S.B., Ashcroft G., Murphy R., Gee I., Poole H., Watson P.M. (2018). Factors influencing patient uptake of an exercise referral scheme: A qualitative study. Health Educ. Res..

[22] Pawson R., Tilley N., Chelimsky E., Shadish W.R. (1997). An Introduction to Scientific Realist Evaluation. Evaluation for the 21st Century: A Handbook.

[23] Oliver E.J., Hanson C.L., Lindsey I.A., Dodd-Reynolds C.J. (2016). Exercise on referral: Evidence and complexity at the nexus of public health and sport policy. Int. J. Sport Policy Politi..

[24] Steele J., Wade M., Polley M., Copeland R.J., Stokes S., Mann S. (2019). The National Referral Database: An initial overview. SportRχiv.

[25] Oliver E.J., Dodd-Reynolds C., Kasim A., Vallis D. (2019). Community-based exercise prescription: Exploring inequalities in engagement and outcomes using the National Referral Database. Open Sci. Framew..

[26] UK Government (2019). Indices of Deprivation. https://www.gov.uk/government/statistics/english-indices-of-deprivation-2019.

[27] Craig C.L., Marshall A.L., Sjöström M., Bauman A.E., Booth M.L., Ainsworth B.E., Pratt M., Ekelund U., Yngve A., Sallis J.F. (2003). International Physical Activity Questionnaire: 12-Country Reliability and Validity. Med. Sci. Sports Exerc..

[28] World Health Organization (2020). Body Mass Index—BMI. https://www.euro.who.int/en/health-topics/disease-prevention/nutrition/a-healthy-lifestyle/body-mass-index-bmi.

[29] Sbaraini A., Carter S.M., Evans R.W., Blinkhorn A. (2011). How to do a grounded theory study: A worked example of a study of dental practices. BMC Med. Res. Methodol..

[30] Morgan D.L., Krueger R.A. (1998). Developing Questions for Focus Groups.

[31] Pawluch D., Neiterman E., De Vries R., Bourgeault I., Dingwall R. (2010). What Is Grounded Theory and Where Does It Come from. The SAGE Handbook of Qualitative Methods in Health Research.

[32] Foley G., Timonen V. (2015). Using grounded theory method to capture and analyze health care experiences. Health Serv. Res..

[33] Timonen V., Foley G., Conlon C. (2018). Challenges when using grounded theory: A pragmatic introduction to doing GT research. Int. J. Qual. Methods.

[34] Public Health England (2018). Local Action on Health Inequalities: Understanding and Reducing Ethnic Inequalities in Health. https://assets.publishing.service.gov.uk/government/uploads/system/uploads/attachment_data/file/730917/local_action_on_health_inequalities.pdf.

[35] Allen A., Sesti F. (2018). Health Inequalities and Women—Addressing Unmet Needs. British Medical Association. https://www.bma.org.uk/media/2116/bma-womens-health-inequalities-report-aug-2018.pdf.

[36] Prince M., Knapp M., Guerchet M., McCrone P., Prina M., Comas-Herrera A., Wittenberg R., Adelaja B., Hu B., King D. (2014). Dementia UK: Update.

[37] National Health Service Digital (2020). Statistics on Obesity, Physical Activity and Diet, England. https://digital.nhs.uk/data-and-information/publications/statistical/statistics-on-obesity-physical-activity-and-diet/england-2020.

[38] Din N.U., Moore G.F., Murphy S., Wilkinson C., Williams N.H. (2014). Health professionals’ perspectives on exercise referral and physical activity promotion in primary care: Findings from a process evaluation of the National Exercise Referral Scheme in Wales. Health Educ. J..

[39] Sport England (2020). Active Lives Adult May 19–20 Coronavirus Report. https://www.sportengland.org/know-your-audience/data/active-lives.

[40] Hanson C.L., Oliver E.J., Dodd-Reynolds C.J., Pearsons A., Kelly P. (2020). A modified Delphi study to gain consensus for a taxonomy to report and classify physical activity referral schemes (PARS). Int. J. Behav. Nutr. Phys. Act..

[41] Bird S.M., Sir David C., Farewell V.T., Harvey G., Tim H., Peter C.S. (2004). Performance indicators: Good, bad, and ugly. J. R. Stat. Soc. Ser. A.

[42] Simplican S.C., Leader G., Kosciulek J., Leahy M. (2015). Defining social inclusion of people with intellectual and developmental disabilities: An ecological model of social networks and community participation. Res. Dev. Disabil..

[43] Gidley J., Hampson G., Wheeler L., Bereded-Samuel E. (2010). From access to success: An integrated approach to quality higher education invormed by social inclusion theory and practice. High. Educ. Policy.

[44] Henderson H., Evans A.B., Allen-Collinson J., Niroshan A.S. (2018). The ‘wild and woolly’ world of exercise referral schemes: Contested interpretations of an exercise as medicine programme. Qual. Res. Sport Exerc. Health.

[45] Glaser B.G. (2007). Constructivist grounded theory?. Hist. Soc. Res. Suppl..

[46] Rouhani S. (2014). Intersectionality-informed quantitative research: A primer. Am. J. Public Health.

[47] Glaser B.G. (1992). Basics of Grounded Theory Analysis: Emergence vs. Forcing.

[48] Benoliel J.Q. (1996). Grounded theory and nursing knowledge. Qual. Health Res..

[49] Sport England (2021). Uniting the Movement. The Five Big Issues—Connecting with Health and Wellbeing. https://www.sportengland.org/why-were-here/uniting-the-movement/what-well-do/connecting-health-and-wellbeing.

