# Supplementary material for: Inequalities and Inclusion in Exercise Referral Schemes: A Mixed-Method Multi-Scheme Analysis
Source: Int J Environ Res Public Health. 2021 Mar 16;18(6):3033. doi: 10.3390/ijerph18063033 (PMC7999569; doi:10.3390/ijerph18063033)
Supplement: Supplementary file 1 [file ijerph-18-03033-s001.pdf]

Supplementary Tables

Table S1: Probability of over/under representation for females at scheme level

**Female (=1)**	**Mean**	**SD**	**2.5% (CI)**	**97.5% (CI)**
Scheme 5001	0.58	0.01	0.56	0.61
Scheme 5002	0.75	0.01	0.74	0.77
Scheme 5026	0.77	0.01	0.75	0.79
Scheme 5036	0.72	0.01	0.71	0.73
Scheme 5056	0.66	0.01	0.64	0.67
Scheme 5063	0.73	0.03	0.68	0.78
Scheme 5072	0.74	0.01	0.72	0.76
Scheme 5089	0.59	0.01	0.57	0.62
Scheme 5108	0.70	0.02	0.66	0.74
Scheme 5115	0.55	0.02	0.52	0.58
Scheme 5119	0.51	0.07	0.38	0.64
Scheme 5131	0.60	0.01	0.58	0.63
Scheme 5144	0.60	0.02	0.56	0.65
Scheme 5156	0.62	0.01	0.59	0.65

Dependent v.: Female

Table S2: Probability of over/under representation for participants of 35 years old or above

**Above35 (=1)**	**Mean**	**SD**	**2.5% (CI)**	**97.5% (CI)**
Scheme 5001	0.90	0.01	0.88	0.91
Scheme 5002	0.86	0.01	0.85	0.87
Scheme 5026	0.87	0.01	0.85	0.88
Scheme 5036	0.85	0.01	0.84	0.86
Scheme 5056	0.84	0.01	0.83	0.85
Scheme 5063	0.81	0.02	0.76	0.85
Scheme 5072	0.85	0.01	0.83	0.86
Scheme 5089	0.90	0.01	0.88	0.91
Scheme 5108	0.84	0.01	0.81	0.87
Scheme 5115	0.91	0.01	0.89	0.92
Scheme 5119	0.86	0.05	0.76	0.94
Scheme 5131	0.81	0.01	0.79	0.83
Scheme 5144	0.84	0.02	0.80	0.87
Scheme 5156	0.94	0.01	0.92	0.95

Dependent v.: Above35. Iterations:1,000

Table S3: Probability of over/under representation for participants from an ethnic minority background at scheme level

EthMinor (=1)	Mean	SD	2.5% (CI)	97.5% (CI)
Scheme 5001	0.44	0.01	0.41	0.47
Scheme 5002	0.74	0.01	0.72	0.75
Scheme 5026	0.76	0.02	0.72	0.79
Scheme 5036	0.56	0.01	0.54	0.58
Scheme 5056	0.63	0.01	0.62	0.65
Scheme 5063	0.68	0.03	0.63	0.74
Scheme 5072	0.69	0.01	0.66	0.71
Scheme 5089	0.06	0.01	0.05	0.07
Scheme 5108	0.31	0.03	0.25	0.37
Scheme 5115	0.01	0.01	0.00	0.03
Scheme 5119	0.00	0.00	0.00	0.00
Scheme 5131	0.44	0.01	0.41	0.47
Scheme 5144	0.06	0.02	0.03	0.09
Scheme 5156	0.35	0.02	0.32	0.38

Dependent v.: EthMinor

Table S4: Probability of over/under representation for participants with IMD decile of 1-5

IMDde	Mean	SD	2.5% (CI)	97.5% (CI)
Scheme 5001	0.79	0.01	0.76	0.81
Scheme 5002	0.93	0.00	0.93	0.94
Scheme 5026	0.89	0.01	0.88	0.91
Scheme 5036	0.59	0.01	0.58	0.61
Scheme 5056	0.54	0.01	0.52	0.55
Scheme 5063	0.93	0.01	0.90	0.96
Scheme 5072	0.98	0.00	0.98	0.99
Scheme 5089	0.73	0.01	0.71	0.75
Scheme 5108	0.12	0.01	0.09	0.15
Scheme 5115	0.75	0.01	0.72	0.78
Scheme 5119	0.12	0.04	0.04	0.22
Scheme 5131	0.91	0.01	0.90	0.92
Scheme 5144	0.08	0.01	0.06	0.11
Scheme 5156	0.91	0.01	0.89	0.93

Dependent v.: HighDeprivation. Iterations: 1,000

Table S5: Scheme completion with interaction terms

Completer (=1)	Mean	SD	2.5% (CI)	97.5% (CI)
Constant	-1.27	0.29	-1.85	-0.71
Female	0.04	0.13	-0.21	0.29
PreSBP	0.00	0.00	0.00	0.00
Scheme 5002-Female	0.27	0.20	-0.13	0.66
Scheme 5144-Female	0.51	1.78	-2.97	4.13
Scheme 5036-Female	0.08	0.15	-0.23	0.38
Scheme 5056-Female	-0.04	0.15	-0.33	0.23
Scheme 5063-Female	-0.16	0.35	-0.86	0.51
Scheme 5072-Female	0.19	0.17	-0.15	0.53
Scheme 5108-Female	-0.52	0.38	-1.28	0.24
Scheme 5156-Female	-0.17	0.76	-1.62	1.34
PreMetMins	0.00	0.00	0.00	0.00
Mental	-0.72	0.18	-1.08	-0.37
LeisureTime	0.15	0.07	0.03	0.28
Above35	0.16	0.22	-0.24	0.59
Age	0.02	0.00	0.02	0.03
Scheme 5002-Above35	0.71	0.32	0.10	1.34
Scheme 5036-Above35	-0.06	0.24	-0.54	0.41
Scheme 5056-Above35	-0.31	0.23	-0.78	0.12
Scheme 5063-Above35	-0.11	0.43	-0.95	0.72
Scheme 5072-Above35	-0.36	0.26	-0.87	0.15
Scheme 5108-Above35	0.22	0.48	-0.71	1.17
IMDde	0.02	0.01	-0.01	0.04
Scheme 5002	-1.14	0.36	-1.86	-0.42
Scheme 5144	-3.26	1.31	-6.39	-1.23
Scheme 5036	-0.35	0.26	-0.87	0.18
Scheme 5056	0.18	0.25	-0.30	0.68
Scheme 5063	0.51	0.51	-0.51	1.46
Scheme 5072	-0.08	0.29	-0.64	0.49
Scheme 5108	0.79	0.52	-0.25	1.82
Scheme 5156	0.12	0.59	-1.00	1.30
PreHR	0.00	0.00	-0.01	0.00
EthMinor	0.00	0.13	-0.25	0.26
Scheme 5002-ethMinor	0.18	0.20	-0.21	0.57
Scheme 5036-ethMinor	-0.08	0.15	-0.38	0.21
Scheme 5056-ethMinor	-0.15	0.14	-0.44	0.13
Scheme 5063-ethMinor	0.11	0.33	-0.54	0.74
Scheme 5072-ethMinor	0.26	0.17	-0.08	0.60
Scheme 5108-ethMinor	0.02	0.37	-0.71	0.73
Scheme 5156-ethMinor	0.18	0.80	-1.40	1.73
PreDBP	0.00	0.00	0.00	0.00
CL1obese	-0.04	0.05	-0.14	0.05
CL2obese	-0.09	0.06	-0.20	0.02
CL3obese	-0.26	0.06	-0.38	-0.13
